# Downregulation of miR-99a/let-7c/miR-125b miRNA cluster predicts clinical outcome in patients with unresected malignant pleural mesothelioma

**DOI:** 10.18632/oncotarget.19800

**Published:** 2017-08-02

**Authors:** Anna Truini, Simona Coco, Ernest Nadal, Carlo Genova, Marco Mora, Maria Giovanna Dal Bello, Irene Vanni, Angela Alama, Erika Rijavec, Federica Biello, Giulia Barletta, Domenico Franco Merlo, Alessandro Valentino, Paola Ferro, Gian Luigi Ravetti, Sara Stigliani, Antonella Vigani, Franco Fedeli, David G. Beer, Silvio Roncella, Francesco Grossi

**Affiliations:** ^1^ Lung Cancer Unit, Ospedale Policlinico San Martino, Genoa, Italy; ^2^ Department of Internal Medicine and Medical Specialties (DIMI), University of Genoa, Ospedale Policlinico San Martino, Genoa, Italy; ^3^ Thoracic Oncology Unit, Department of Medical Oncology, Catalan Institute of Oncology (ICO), L'Hospitalet del Llobregat, Barcelona, Spain; ^4^ Molecular Mechanisms and Experimental Therapeutics Program, Bellvitge Biomedical Research Institute (IDIBELL), L’Hospitalet de Llobregat, Barcelona, Spain; ^5^ Section of Thoracic Surgery, Department of Surgery, University of Michigan Medical School, Ann Arbor, Michigan, USA; ^6^ Department of Pathology, Ospedale Policlinico San Martino, Genoa, Italy; ^7^ Research and Statistics Infrastructure, Azienda Unità Sanitaria Locale di Reggio Emilia - IRCCS, Institute for Advanced Technologies and HealthCare Protocols in Oncology, Reggio Emilia, Italy; ^8^ Division of Histopathology and Cytopathology, ASL5, La Spezia, Italy; ^9^ UOS Physiopathology of Human Reproduction, Ospedale Policlinico San Martino, Genoa, Italy; ^10^ Division of Oncology, ASL5, La Spezia, Italy

**Keywords:** malignant pleural mesothelioma, unresectable tumors, miRNA signature, miR-99a/let-7c/miR-125b miRNAs cluster, prognostic biomarkers

## Abstract

Malignant pleural mesothelioma (MPM) is an aggressive tumor with a dismal overall survival (OS) and to date no molecular markers are available to guide patient management. This study aimed to identify a prognostic miRNA signature in MPM patients who did not undergo tumor resection. Whole miRNA profiling using a microarray platform was performed using biopsies on 27 unresected MPM patients with distinct clinical outcome: 15 patients had short survival (OS<12 months) and 12 patients had long survival (OS>36 months). Three prognostic miRNAs (mir-99a, let-7c, and miR-125b) encoded at the same cluster (21q21) were selected for further validation and tested on publicly available miRNA sequencing data from 72 MPM patients with survival data. A risk model was built based on these 3 miRNAs that was validated by quantitative PCR in an independent set of 30 MPM patients. High-risk patients had shorter median OS (7.6 months) as compared with low-risk patients (median not reached). In the multivariate Cox model, a high-risk score was independently associated with shorter OS (HR=3.14; 95% CI, 1.18–8.34; P=0.022). Our study identified that the downregulation of the miR-99a/let-7/miR-125b miRNA cluster predicts poor outcome in unresected MPM.

## INTRODUCTION

Malignant Pleural Mesothelioma (MPM) originates from mesothelial cells that cover the pleura. The most common cause of this disease is the exposure to the asbestos fiber; indeed, MPM is predominant in areas where there are heavy or chemical industries, shipyards and oil refineries known to be activities related to asbestos usage. MPM is characterized by a long latency and its incidence is expected to increase in some industrialized countries over the next 5-10 years [1]. Long-term survival rate of patients with MPM is generally poor with a median overall survival (OS) of 9-11 months [2].

MPM can be histologically classified into three major subtypes: epithelioid, sarcomatoid and biphasic. Most MPM patients are considered not suitable for a multimodal therapeutic approach and the majority of patients are treated with palliative chemotherapy. Patients are deemed suitable for radical treatment with curative intent if the following criteria are met: epithelioid histology, absence of lymph nodal involvement, technically resectable disease, and ability to tolerate major pulmonary surgery. In Europe, multimodal therapy including induction with platinum-based chemotherapy, extra-pleural pneumonectomy (EPP) and radiation therapy is generally performed in selected patients in specialized hospitals. As this technique is associated with relevant perioperative morbidity and mortality, another surgical approach, pleurectomy with decortication (P/D), emerged as a lung-sparing procedure suitable for the radical treatment of patients with good performance status and early stage of disease [3]. Globally, while several studies have been conducted with the aim of addressing harms and benefits of surgery in MPM, its current role remains controversial, mostly due to difficulties in patient enrollment and different expertise across surgical departments; notably, a feasibility trial designed to clarify the role of P/D (MARS2) is currently ongoing [4]. Cisplatin with pemetrexed showed longer median OS as compared with cisplatin alone and is considered the standard of care for patients with unresectable MPM [5]. In the MAPS trial, the addition of bevacizumab to cisplatin and pemetrexed demonstrated a significant increase in the OS compared with the standard treatment [6]. There is no established second-line standard of care, although single-agent regimens with gemcitabine or vinorelbine represent a suitable approach, as well as re-challenge with pemetrexed-based chemotherapy in case of good and sustained response during first-line treatment [3].

Among the MPM patients who could not undergo surgery with radical intent, there is a relatively small subgroup whose OS is greater than 3 years; therefore, taking into account this observation, we interrogated whether specific markers were able to predict prognosis and define which patients might benefit from aggressive approaches, eventually including multi-modality treatments. To date, numerous studies reported how expression level of specific miRNAs, small noncoding RNA involved in gene expression regulation, is able to precisely predict prognosis in MPM [7–12]. Recently, Kirschner and colleagues reported the first miRNA prognostic signature in surgically resected MPM patients [13]. Specifically, the authors identified 6 differentially-expressed miRNAs between long and short survivors in a cohort of patients who underwent surgery. However, a significant limitation of this study is represented by the low number of MPM patients who are deemed to be adequate candidates for surgery.

We hypothesized that miRNA expression profiling might identify relevant markers associated with clinical outcome in patients with unresectable MPM, which represent the majority of cases observed in clinical practice. In the present study, we first performed a miRNA microarray expression profiling using formalin-fixed paraffin embedded (FFPE) tumor biopsy samples from patients affected by MPM who did not undergo surgery and then selected miRNAs with prognostic value were tested in an independent cohort of patients by quantitative PCR (qPCR).

## RESULTS

### Patient characteristics

Tumor biopsy samples from patients with unresectable MPM were divided in two independent sets: training set (TS) and validation set (VS), consisting of 27 and 30 samples respectively. Clinical and demographic characteristics of patients included are shown in Table 1. There were no significant differences according to age, gender, smoking history, asbestos exposure or histopathological subtype between both sets. About half of patients in each set were considered long-term survivors.

**Table 1:** Characteristics of patients included in the training set and the validation set

Characteristics	Training set	Validation set	All	*p*-value
	(n=27)	(n=30)	(n=57)	
**Age- years**				
Median (SD)	67.9 (± 6.5)	69.5 (± 8.5)	68.7 (± 7.6)	0.540†
**Sex –*n* (%)**				
Male	22 (81%)	25 (83%)	47 (82.5%)	
Female	5 (19%)	5 (17%)	10 (17.5%)	0.854‡
**Smoking –*n* (%)**				
Smokers	18 (67%)	19 (63%)	37 (65%)	
Never smokers	9 (33%)	9 (30%)	18 (31.5%)	
Unknown	0 (0%)	2 (7%)	2 (3.5%)	0.391‡
**Asbestos exposure –*n* (%)**				
Yes	22 (81%)	20 (67%)	42 (74%)	
Unknown	5 (19%)	10 (33%)	15 (26%)	0.205‡
**Histological subtype –*n* (%)**				
Epithelioid	20 (74%)	21 (70%)	41 (72%)	
Sarcomatoid	3 (11%)	4 (13%)	7 (12%)	
Biphasic	3 (11%)	3 (10%)	6 (10.5%)	
Desmoplastic	0 (0%)	2 (7%)	2 (3.5%)	
Not typified	1 (4%)	0 (0%)	1 (2%)	0.555‡
**Outcome –*n* (%)**				
Short survivor	15 (55.5%)	16 (53%)	31 (54%)	
Long survivor	12 (44.5%)	14 (47%)	26 (46%)	0.866‡

†, Student’s t-test; ‡, Chi-square test.

### MiRNA microarray profiling yielded differentially-expressed miRNAs

**Figure 1:**
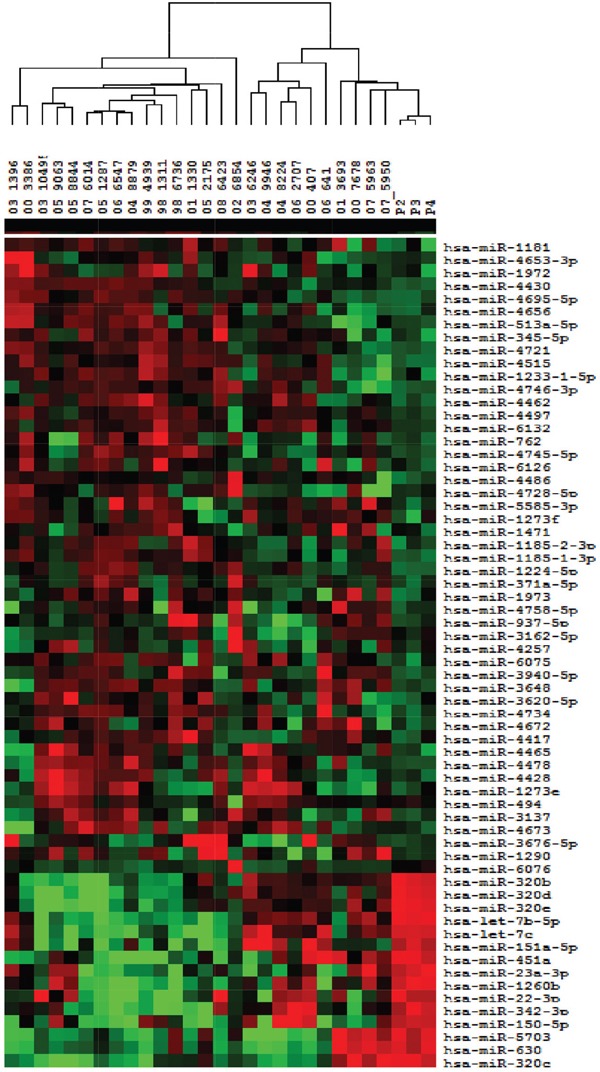
Supervised clustering of differentially expressed miRNAs among mesothelioma and nonmalignant pleural samples Samples (26 MPM tumors and 3 nonmalignant pleura) are depicted in columns and miRNAs in rows. Substantially elevated (red) or decreased (green) expression of the miRNAs is observed for individual tumors. Samples and arrays were mean-centered, centroid linkage clustering using Cluster *v.*3.0 and visualization using Tree-View software.

All 27 tumor samples corresponding to the TS and 4 nonmalignant pleural samples were hybridized to the miRNA microarray, however two cases (1 long survivor and 1 normal control) failed the quality control step and were excluded from the analysis (Supplementary Figure 1). Overall, the mean number of miRNAs expressed per sample was 297 and 116 miRNAs were expressed in all samples. MiRNAs with more than 30% missing data across all samples were filtered out and a total of 259 human miRNAs were retained in the final analysis. Among the final 259 miRNAs, 91 miRNAs were found differentially expressed in 26 MPMs as compared to 3 nonmalignant pleura at p-value<0.05 (Mann-Whitney U test). Specifically, 58 miRNAs were significantly up-regulated (fold change>2), whereas 33 were down-regulated in tumor tissues (fold change<0.5), as shown in Table 2. Supervised clustering based on 64 differentially expressed miRNAs at p-value<0.01 is shown in Figure 1.

**Table 2:** MiRNAs differentially expressed between mesothelioma tumor and nonmalignant pleura

ID	Average MPM	Average pleura	Fold change	P-value
hsa-miR-4486	0.25	−2.87	8.66	0.0052
hsa-miR-4497	4.77	2.25	5.76	0.0099
hsa-miR-1181	0.71	−1.66	5.17	0.0052
hsa-miR-1273e	−0.68	−3.03	5.12	0.0081
hsa-miR-4465	3.11	0.77	5.04	0.0052
hsa-miR-4430	2.91	0.64	4.82	0.0052
hsa-miR-513b	0.03	−2.06	4.25	0.0149
hsa-miR-4653-3p	2.53	0.45	4.23	0.0052
hsa-miR-1224-5p	3.03	0.96	4.21	0.0052
hsa-miR-4462	0.07	−1.94	4.02	0.0052
hsa-miR-4428	2.57	0.60	3.92	0.0081
hsa-miR-3676-5p	5.89	3.95	3.83	0.0052
hsa-miR-6132	3.27	1.44	3.57	0.0099
hsa-miR-4734	0.20	−1.62	3.51	0.0065
hsa-miR-345-5p	−1.43	−3.23	3.47	0.0052
hsa-miR-4656	0.54	−1.23	3.42	0.0052
hsa-miR-1273f	1.14	−0.63	3.42	0.0099
hsa-miR-1972	2.04	0.31	3.33	0.0065
hsa-miR-6075	0.46	−1.27	3.30	0.0065
hsa-miR-4721	3.70	1.98	3.29	0.0052
hsa-miR-1185-2-3p	0.49	−1.14	3.10	0.0052
hsa-miR-1233-1-5p	0.13	−1.49	3.08	0.0081
hsa-miR-4515	0.96	−0.63	3.02	0.0052
hsa-miR-197-5p	−0.40	−1.98	2.99	0.0149
hsa-miR-4478	1.23	−0.35	2.99	0.0052
hsa-miR-513a-5p	1.27	−0.30	2.97	0.0052
hsa-miR-1185-1-3p	1.89	0.33	2.95	0.0052
hsa-miR-4695-5p	1.18	−0.38	2.95	0.0052
hsa-miR-4758-5p	−0.25	−1.80	2.94	0.0052
hsa-miR-6076	2.01	0.48	2.89	0.0052
hsa-miR-3620-5p	0.09	−1.43	2.87	0.0065
hsa-miR-1471	1.59	0.10	2.82	0.0099
hsa-miR-4746-3p	1.63	0.15	2.78	0.0052
hsa-miR-4257	1.42	−0.05	2.78	0.0065
hsa-miR-762	4.21	2.74	2.76	0.0052
hsa-miR-1973	5.05	3.60	2.74	0.0052
hsa-miR-371a-5p	0.59	−0.85	2.71	0.0052
hsa-miR-4672	1.96	0.53	2.70	0.0081
hsa-miR-3940-5p	2.89	1.46	2.69	0.0052
hsa-miR-21-5p	−1.05	−2.41	2.56	0.0219
hsa-miR-494	6.55	5.19	2.55	0.0099
hsa-miR-3648	1.53	0.19	2.53	0.0081
hsa-miR-4322	−0.23	−1.56	2.51	0.0149
hsa-miR-3137	0.92	−0.37	2.44	0.0052
hsa-miR-6126	1.94	0.69	2.39	0.0065
hsa-miR-5585-3p	1.59	0.35	2.37	0.0081
hsa-miR-4745-5p	1.08	−0.09	2.24	0.0052
hsa-miR-3188	0.72	−0.44	2.23	0.0122
hsa-miR-671-5p	1.98	0.84	2.21	0.0378
hsa-miR-4728-5p	1.73	0.59	2.20	0.0081
hsa-miR-4417	0.75	−0.37	2.18	0.0065
hsa-miR-937-5p	2.87	1.76	2.16	0.0052
hsa-miR-574-5p	2.58	1.47	2.16	0.0450
hsa-miR-135a-3p	0.55	−0.55	2.14	0.0149
hsa-miR-3162-5p	4.62	3.54	2.11	0.0099
hsa-miR-4673	−0.49	−1.54	2.08	0.0099
hsa-miR-1290	−0.57	−1.61	2.05	0.0099
hsa-miR-3917	−0.40	−1.41	2.01	0.0219
hsa-miR-4778-5p	−0.84	0.21	0.48	0.0264
hsa-miR-6090	6.36	7.42	0.48	0.0264
hsa-let-7i-5p	1.89	2.98	0.47	0.0317
hsa-miR-93-5p	−1.52	−0.34	0.44	0.0264
hsa-let-7e-5p	1.11	2.31	0.44	0.0317
hsa-miR-483-5p	0.42	1.65	0.43	0.0122
hsa-miR-378a-3p	−0.48	0.77	0.42	0.0181
hsa-let-7a-5p	3.79	5.09	0.41	0.0219
hsa-let-7g-5p	0.79	2.22	0.37	0.0181
hsa-let-7d-5p	0.59	2.07	0.36	0.0149
hsa-miR-151a-5p	−1.33	0.25	0.34	0.0099
hsa-miR-125b-5p	1.91	3.60	0.31	0.0219
hsa-miR-342-3p	−1.51	0.20	0.31	0.0099
hsa-miR-5703	1.10	2.81	0.30	0.0081
hsa-miR-199a-3p	0.42	2.14	0.30	0.0264
hsa-miR-320b	0.44	2.18	0.30	0.0052
hsa-miR-630	1.66	3.42	0.30	0.0081
hsa-miR-23a-3p	1.22	3.06	0.28	0.0052
hsa-miR-15b-5p	0.38	2.23	0.28	0.0181
hsa-miR-199a-5p	−1.50	0.43	0.26	0.0378
hsa-miR-25-3p	−1.06	0.93	0.25	0.0181
hsa-miR-320d	0.14	2.16	0.25	0.0052
hsa-miR-195-5p	−0.88	1.19	0.24	0.0264
hsa-miR-92a-3p	−1.32	0.75	0.24	0.0264
hsa-miR-320e	−0.44	1.66	0.23	0.0052
hsa-miR-320c	1.26	3.54	0.21	0.0081
hsa-let-7b-5p	3.88	6.26	0.19	0.0052
hsa-let-7c	1.79	4.25	0.18	0.0065
hsa-miR-22-3p	−1.27	1.42	0.16	0.0099
hsa-miR-99a-5p	−1.19	1.69	0.14	0.0219
hsa-miR-1260b	−0.98	2.09	0.12	0.0052
hsa-miR-150-5p	−1.43	1.77	0.11	0.0099
hsa-miR-451a	0.13	5.10	0.03	0.0052

List of the 91 miRNAs found differentially expressed between 26 MPMs and 3 nonmalignant pleura (p-value<0.05) by microarray analysis. Specifically, 58 miRNAs were significantly up-regulated (fold change>1.5), whereas 33 were down-regulated in tumor tissues (fold-change<0.5).

### Hierarchical clustering yielded two major clusters associated with clinical outcome

**Figure 2:**
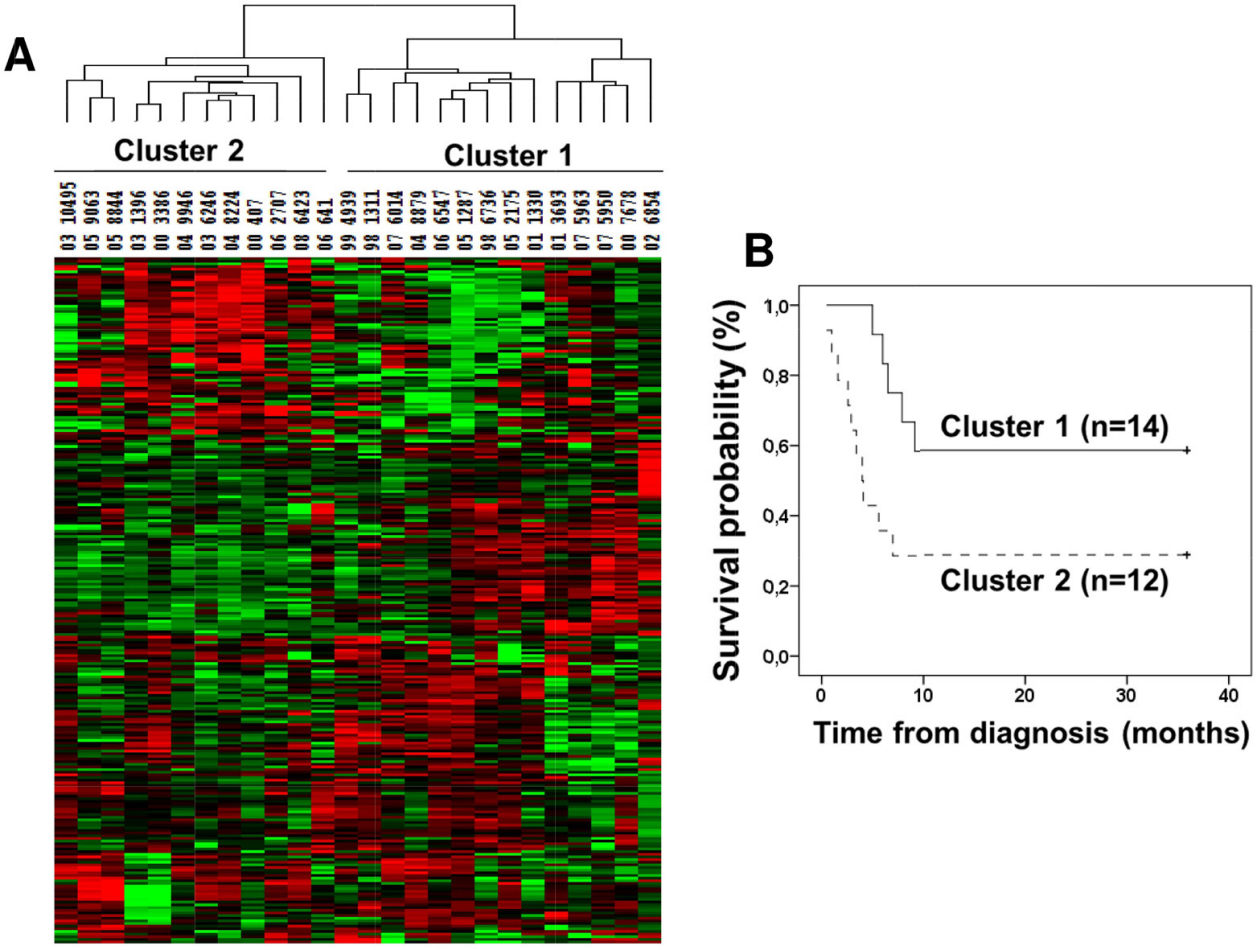
Unsupervised hierarchical clustering of miRNA expression in mesothelioma tumors **(A)** Two major clusters of tumors were identified by unsupervised clustering analysis based on 259 miRNAs expressed in all tumors. **(B)** Kaplan-Meier plot of overall survival according to the cluster subgroups.

In the TS, hierarchical clustering analysis based on 259 miRNAs yielded two major tumor clusters (Figure 2A). There was no statistically significant difference in age, gender or histological subtype among patients classified according to these clusters (data not shown). Interestingly, patients classified in cluster 2 had significantly shorter median OS (7 months) as compared with patients categorized in cluster 1 (median OS not reached, Log-rank p=0.035, Figure 2B).

To identify individual miRNAs significantly associated with clinical outcome, we performed class-comparison analysis among short and long survivors. We identified 35 differentially-expressed miRNAs at a t-test p-value<0.05 among short and long survivors that were also associated with clinical outcome in the univariate analysis (Cox regression p-value<0.05, Table 3).

**Table 3:** MiRNAs significantly associated with survival

ID	Average SS	Average LS	ttest p-value	Fold Change	Cox HR	Cox p-value	TCGA dataset validation
**miR-99a-5p**	−2.07	−0.15	0.0031	0.26	0.42	0.0014	**yes**
**let-7c**	1.28	2.37	0.0066	0.47	0.32	0.0014	**yes**
**miR-125b-5p**	1.31	2.62	0.0084	0.40	0.41	0.0010	**yes**
miR-26b-5p	−1.35	−0.13	0.0070	0.43	0.45	0.0022	**yes**
miR-371a-5p	0.79	0.36	0.0120	1.35	42.57	0.0010	**yes**
miR-23b-3p	−0.28	1.00	0.0201	0.41	0.53	0.0036	**yes**
miR-107	−0.66	0.01	0.0275	0.63	0.27	0.0040	**yes**
miR-26a-5p	0.06	1.02	0.0370	0.51	0.50	0.0041	**yes**
miR-30b-5p	−2.13	−1.10	0.0126	0.49	0.46	0.0045	**yes**
miR-1185-1-3p	2.07	1.69	0.0107	1.30	11.73	0.0050	**yes**
miR-29c-3p	−1.21	−0.26	0.0497	0.52	0.56	0.0120	**yes**
miR-342-3p	−1.82	−1.14	0.0333	0.62	0.40	0.0232	**yes**
miR-652-5p	−0.84	−1.28	0.0165	1.36	17.49	0.0010	opposite
miR-1236-5p	0.69	0.37	0.0010	1.25	52.14	0.0020	no
miR-130a-3p	−0.37	1.08	0.0018	0.37	0.40	0.0010	no
miR-937-5p	3.09	2.61	0.0023	1.40	12.21	0.0007	no
miR-1224-5p	3.25	2.78	0.0033	1.39	35.63	0.0010	no
miR-197-5p	5.23	4.84	0.0054	1.31	11.85	0.0018	no
miR-25-3p	−1.43	−0.63	0.0054	0.57	0.13	0.0017	no
let-7d-5p	0.14	1.11	0.0085	0.51	0.23	0.0002	no
miR-642a-3p	5.53	5.11	0.0085	1.34	22.97	0.0020	no
miR-320e	−0.71	−0.13	0.0142	0.67	0.27	0.0094	no
miR-3137	1.10	0.70	0.0144	1.33	17.53	0.0020	no
miR-16-5p	1.33	2.28	0.0153	0.52	0.2917	0.0002	no
miR-151a-5p	−1.71	−0.88	0.0155	0.57	0.34	0.0030	no
miR-3162-5p	4.92	4.27	0.0195	1.57	14.91	0.0020	no
miR-1229-5p	3.40	3.02	0.0289	1.30	6.37	0.0070	no
let-7b-5p	3.60	4.21	0.0302	0.66	0.24	0.0023	no
miR-939-5p	3.06	2.72	0.0329	1.27	17.76	0.0014	no
let-7a-5p	3.49	4.15	0.0373	0.63	0.33	0.0049	no
miR-671-5p	2.31	1.61	0.0379	1.62	7.4268	0.0117	no
miR-15b-5p	0.04	0.77	0.0384	0.60	0.40	0.0075	no
miR-1185-2-3p	0.65	0.31	0.0394	1.27	7.61	0.0130	no
let-7f-5p	2.35	3.01	0.0446	0.64	0.31	0.0076	no
miR-22-3p	0.85	1.65	0.0469	0.58	0.47	0.0232	no

List of miRNAs significantly associated with clinical outcome in the training set. Fold-change was calculated as mean value in short survivors (SS) in respect to long survivors (LS). The last column shows whether the prognostic value of each miRNA was validated using TCGA data.

### A 3-miRNA prognostic signature was validated using TCGA data

**Figure 3:**
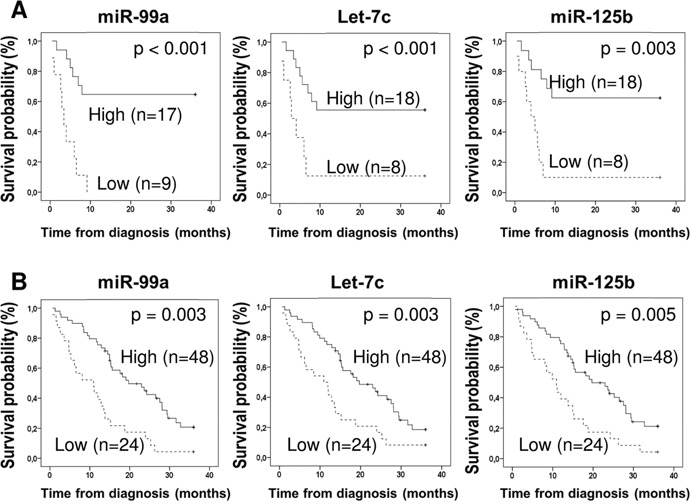
Prognostic value of miR-99a, let-7c, and miR-125b cluster expression in two independent cohorts of mesothelioma Patients with tumors with low expression of these miRNAs (low third) had significantly shorter OS compared with tumors with high expression (bottom two thirds) in: **(A)** training set (n=26) and **(B)** TCGA set (n=72).

In order to validate our findings and to assess the prognostic value of these 35 miRNAs in an independent dataset, we used publicly available miRNA sequencing data from 72 MPM patients deposited in TCGA. Only 12 miRNAs remained statistically associated with OS (Table 3 and Supplementary Table 1). Interestingly, the top 3 prognostic miRNAs (miR-99a, let-7c, and miR-125b) were structurally associated by their genomic location on the long arm of chromosome 21 (21q21.1) and selected for further validations. In the TS, patients with low expression of miR-99a, let-7c, and miR-125b had significantly shorter median OS (2.9, 2.9 and 3.4 months, respectively) as compared to patients with high expression of these miRNAs (median OS not reached, Figure 3A). Accordingly, in the TCGA dataset, patients with low expression of miR-99a, let-7c, and miR-125b had significantly shorter median OS (10.9 months for all three markers) as compared to patients with high expression (19.7, 19.7, and 24.1 months, Figure 3B).

The expression of miR-99a, let-7c, and miR-125b was assessed using qPCR in the TS and a close correlation was observed among the expression values generated by microarray and qPCR (Pearson r coefficient: 0.55; 0.71 and 0.42 respectively, Supplementary Figure 2). In order to explore the hypothetical biological effect of these 3 miRNAs, we carried out a pathway enrichment analysis based on the validated gene targets of miR-99a, let-7c, and miR-125b. This analysis revealed that these miRNAs are significantly associated with pathways relevant in mesothelioma such as cell cycle, pluripotency of stem cells, HIF-1, ErbB and AMPK signaling pathways (Supplementary Table 2).

### Validation of a miRNA prognostic signature by qPCR in an independent cohort

In the validation set, we observed that miR-99a, let-7c, and miR-125b expression did not correlate with the histological type of mesothelioma (Mann-Whitney p>0.05), whereas their expression values were significantly higher in long-surviving patients (Mann-Whitney p<0.05, Supplementary Figure 3).

**Figure 4:**
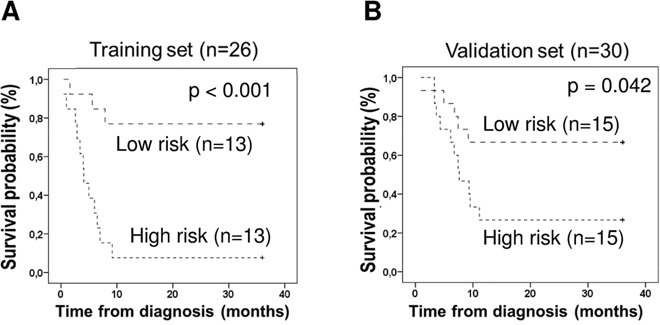
Kaplan-Meier plots of overall survival according to risk index based on the expression of mir-99a, let-7c, and miR-125b Patients whose tumors had high-risk score show significantly shorter median OS as compared with patients with low-risk in two independent cohorts: **(A)** training set and **(B)** validation set.

A miRNA signature was built based on the expression of miR-99a, let-7c, and miR-125b by calculating a risk score based on the sum of the expression of these miRNAs in the VS, weighted by the corresponding regression coefficients (β) derived from the Cox regression analysis in the TS, as previously reported [14]. The median risk score value was used as cutoff to classify the patients into high-risk or low-risk. In this VS, patients with a high-risk score had a significantly shorter median OS (7.6 months; 95% confidence interval, CI, 4.4–10.9) as compared with low-risk patients (median not reached, log-rank p=0.042, Figure 4). The OS rates at 24 months for high- and low-risk patients were 27.7% ± 11.4 and 66.7% ± 11.2, respectively. In the multivariate Cox model (adjusted by age and histological subtype) a high-risk score remained as an independent prognostic marker for OS (Hazard Ratio (HR) =3.14; 95% CI, 1.18–8.34; p=0.022). The prognostic value of these 3 miRNAs (miR-99a, let-7c, and miR-125b) encoded at the 21q21 was therefore validated in an independent set of MPM.

### Copy number analysis of miR-99a/let-7c/miR-125b locus

Copy number variation (CNV) analysis of the miR-99a/let-7c/miR-125b locus (21q21.1) was performed using droplet digital PCR (ddPCR) on 44 out of 57 patients enrolled (16/27 TS and 28/30 VS) divided as follows: 17 long survivors (LS) and 27 short survivors (SS) patients. In particular, the miR-99a/let-7c/miR-125b locus data was normalized using two genes: RNA binding motif protein 11 (*RBM11*) targeting the centromere locus on chromosome 21 (to discern 21 monosomy) and adaptor-related protein complex 3, beta 1 subunit (*AP3B1*) mapping on 5q14.1. The CNV analysis reported 21q21.1 locus mean values in the two MPM sub-populations as follows: 1.91 (range: 1.62-2.27) and 1.88 (range: 1.19-2.31) in LS and SS patients, respectively (Supplementary Figure 4). In particular, two SS patients showed a copy number ratio lower than 1.5, however when data were normalized based on *RBM11* that targets the centromere, the obtained copy number ratio was diploid (1.97 and 2.35). These results suggested a potential imbalance between the two chromosomes (21 monosomy or 5 extra-copies) rather than a loss of a copy of the locus.

## DISCUSSION

MiRNA expression profiling represents a powerful tool for identifying prognostic markers in cancer, including MPM [10]. However, to the best of our knowledge, no miRNA signature has ever been identified for predicting prognosis in unresected MPM patients. Toward this aim, in the present study we screened the whole miRNA profile in a cohort of 26 MPM patients who did not undergo radical surgery, in order to identify novel prognostic markers. The analysis identified 46 miRNAs differentially expressed in MPM specimens, some of which had been previously reported in MPM, such as downregulation of let-7 in MDM2 positive MPM tumors [15], and low expression of miRNA23a-3p in a subgroup of MPM long survivors after surgery [13].

In order to investigate miRNAs with prognostic value, the 26 MPM patients were divided based on whether they had short or long survival. Class-comparison analysis of miRNA expression among short and long survivors yielded 35 differentially-expressed miRNAs, and 32 out of 35 were significantly associated with clinical outcome in the univariate analysis (p-value<0.05). The prognostic value of 12 miRNAs out of these 35 was validated in an independent dataset composed of 72 MPM patients using publicly available miRNA sequencing data from TCGA.

Notably, the top three prognostic miRNAs (miR-99a, let-7c, and miR-125b) among those 12 validated miRNAs, were structurally associated by their genomic location on the long arm of chromosome 21 (21q21.1).

These 3 miRNAs might play a tumor suppressor role, as they have been found to be down-regulated in distinct tumor types. In particular, their down-modulation has been described in lung cancer linked to a homozygous deletion at 21q21.1 [16], although in our cohort of samples we did not find any CNVs at the miR-99a/let-7c/miR-125b cluster. Interestingly, miR-99a is among the most frequently down-regulated miRNAs in cancer, and its low expression has been linked to increased proliferation in different tumor types such as head and neck carcinoma and cervical carcinoma, as well as lung cancer, breast cancer and melanoma [17–20]. Likewise, let-7c is down-regulated in a variety of cancer types such as lung and prostate cancer, melanoma and Wilms’ tumors; indeed, lack of let-7c expression is associated with increased cell proliferation in both prostate and lung cancer [21, 22]. Similarly, low expression of miR-125b has been described in epithelial ovarian cancer and oral cavity squamous cell carcinoma [23, 24]. In addition, two recent studies showed concomitant downregulation of the miR-99a/let-7c/miR-125b cluster both in cholangiocarcinoma and prostate cancer [25, 26]. In particular, downregulation of this cluster has been linked to inflammation through the activation of IL6/STAT3 pathway [26]. Association between inflammation and MPM has already been investigated, and evidence suggests that activation of IL6 plays a role in cell proliferation and chemo-resistance [27, 28]. A further potential demonstration of the role of the miR-99a/let-7c/miR-125b cluster as a tumor suppressor might be found in patients affected by Down syndrome, a genetic disorder characterized by chromosome 21 trisomy [29]. In this regard, Forés-Martos and colleagues hypothesized that the low incidence rate of solid tumors in subjects with Down syndrome might be related to the gain of copies of this miRNA cluster, located on chromosome 21 [19].

A miRNA signature was built based on the expression of miR-99a, let-7c, and miR-125b, and tested in an independent set of MPM samples in which patients were classified as high-risk or low-risk according to their median risk score value. Interestingly, patients with high-risk had a shorter OS as compared to low-risk patients, and in the multivariate Cox model a high-risk score remained as an independent prognostic marker for OS.

Currently, a number of miRNAs have been correlated with prognosis in MPM patients [30–32]; in particular, Kirschner and colleagues and Lin and colleagues recently proposed the first prognostic miRNA signatures of surgically resected MPM patients [13, 32]. The lack of overlap between our miRNA signature and their results might be explained by differences in the study population and in the methods used to measure miRNA expression and to normalize the data. Specifically, Kirschner and colleagues identified a miRNA signature in a cohort of patients who underwent EPP [13]; such signature was then validated in a group of patients subjected to P/D performed within a single institution. In contrast, our prognostic miRNA signature was developed in a cohort of unresected patients, and was identified by a high-throughput approach and then validated in two independent cohorts of patients. These differences underscore the need for building collaborative consortiums able to collect large cohorts of patients with mesothelioma to identify and validate robust markers for early diagnosis and prognosis. Overall, our results suggest that low expression of the 21q21.1 miRNA cluster could have a prognostic significance in unresected patients. However, a limitation of our study is the relatively low number of patients included in the VS mainly due to the fact that collecting samples from unresected MPM patients with long-term survival is challenging.

In conclusion, to the best of our knowledge, this is the first prognostic signature able to predict survival in patients who have not undergone surgery for MPM. Furthermore, identifying patients with different prognosis might guide clinical decisions whether to propose more aggressive or more conservative therapeutic approaches. Additionally, patients with an unfavorable miRNA signature could also benefit from novel therapeutic strategies in the future such as miRNA-based treatments, although further basic research is needed to verify the efficient translation of these approaches.

## MATERIALS AND METHODS

### Patient enrollment

In the present study, a total number of 27 patients diagnosed with MPM at the Istituto Nazionale per la Ricerca sul Cancro, Genova, Italy were enrolled as TS. In addition, 30 patients with clinically confirmed diagnosis of MPM were enrolled at San Andrea Hospital, La Spezia, Italy as VS. According to the clinical outcome, patients were divided into two groups: *i)* patients with SS, who died within 12 months from the diagnosis (TS: 15; VS: 16) and *ii)* LS patients whose OS was greater than 36 months (TS: 12; VS: 14). All patients who either underwent surgery or were older than 80 years at diagnosis were not eligible and hence excluded from the study.

Four additional biopsies of nonmalignant pleura obtained from healthy individuals were processed as a normal control (NC). The non-neoplastic controls of pleural tissue were obtained from surgical specimens of lobectomy performed for non-small cell lung cancer (NSCLC, 3 adenocarcinoma and 1 squamous cell carcinoma), in which portions of parietal pleura were present. At gross examination, all NSCLCs did not show pleural invasion and histologic sections of all pleural-tissue samples were reviewed by the pathologist in order to rule out the presence of neoplastic cells.

The present study was approved by the institutional ethics committees (Istituto Nazionale per la Ricerca sul Cancro, Genova, Italy n. OT11.001, May 25, 2012 for TS; Ethics Committee of the Liguria Region P.R. 207REG2014, September 23, 2014 for VS), and conducted according to the provisions of the Declaration of Helsinki. The data were analyzed anonymously.

### RNA and genomic DNA (gDNA) extraction

For each case, the FFPE biopsy tumor block was reviewed by the pathologist to check for the presence of adequate tumor cell content (>50%). Total RNA, including the miRNA fraction, was isolated from two FFPE macro-dissected tissue sections (10 μm) using the Recover All Total Nucleic Acid Isolation Kit for FFPE Tissue (ThermoFisher Scientific, Wilmington, DE, USA), including a RNase-free DNase I treatment. RNA quantity and quality were assessed by NanoDrop ND-1000 (ThermoFisher Scientific) and 2100 Bioanalyzer instrument using RNA 6000 Nano Kit (Agilent Technologies, Santa Clara, CA, USA). All the samples that reported at least 150 ng of total RNA were defined suitable for further analyses. For 44 out of 57 patients enrolled in the study, two additional tumor sections (10 μm) were available to isolate gDNA using GeneRead DNA FFPE Kit (Qiagen, Hilden, Germany) that was quantified by Qubit® 2.0 Fluorometer (ThermoFisher Scientific).

### Microarray analysis

MiRNA profiling was performed on 27 MPMs from TS (15 SS and 12 LS) and 4 nonmalignant pleural samples. Total RNA containing miRNA was processed following the ‘miRNA Microarray System protocol’ *v.*2.4 (Agilent Technologies), as previously described [33]. Briefly, 100 ng of total RNA and an appropriate dilution of Spike-in controls underwent dephosphorylation and labeling step with Cy3CTP and purification using Micro Bio-Spin™ P-6 Gel Columns (Bio-Rad, Hercules, CA, USA). Then, each sample was hybridized on Human miRNA microarray slide 8×60K (miRBase Release 19.0) (Agilent Technologies), including 2006 human miRNAs. After washing, the slides were scanned by G2565CA scanner (Agilent Technologies) and data were extracted by Feature Extraction software *v.*10 (Agilent Technologies). Microarray expression data were normalized using GeneSpring software *v*.12.6 (Agilent Technologies). Microarray raw data have been deposited in Gene Expression Omnibus (http://www.ncbi.nlm.nih.gov/geo/; GEO number: GSE92636) [34]. Missing values corresponding to each unexpressed miRNA were filled using the minimum value and then subtracting 0.5.

### Validation of selected miRNAs using publicly available data

Clinical outcome and miRNAseq data were available from 72 patients with MPM included in the TCGA-Mesothelioma project, and these data were downloaded.

### Analysis of genes targeted by select miRNAs

Validated genes targeted by select miRNAs were obtained from mirTarBase database [35]. An enriched pathway analysis was performed using DAVID functional annotation bioinformatics microarray analysis [36].

### Validation of selected miRNAs by qPCR

Three selected miRNAs were then validated in a different set of tissue samples consisting of 30 patients from VS (16 SS and 14 LS) and 4 NC by qPCR using TaqMan® miRNA Assays (hsa-miR-99a #000435; hsa-let-7c #000379; hsa-miR-125b #000449; U6 snRNA #001973 as reference miRNA; ThermoFisher Scientific). Briefly, 10 ng of total RNA were retro-transcripted into cDNA with a specific primer using TaqMan® MiRNA Reverse Transcription Kit (ThermoFisher Scientific). Then each selected miRNA was amplified in duplicate on RealPlex^2^ system (Eppendorf, Hamburg, Germany) using qPCR TaqMan® Universal Master Mix II, no Uracil-N glycoslyase (ThermoFisher Scientific) and normalized against U6 snRNA. The relative expression compared to the NC was determined using the equation 2^−ΔΔCT^. A patient's risk score was calculated as the sum of the expression levels of the 3 selected prognostic miRNAs in the VS, weighted by the corresponding regression coefficients (β) derived from the Cox regression analysis in the TS, as previously reported [14]. The risk score was used to classify patients into high- or low-risk groups, with a high-risk score indicating poorer survival. In the VS, the median of the risk score was used as the cutoff value.

### Copy number determination of the miR-99a/let-7c/miR-125b locus

The CNV study of the miR-99a/let-7c/miR-125b locus (21q21.1) was assessed in both TS and VS tumor samples by ddPCR according to the multiplexing strategy (three targets quantified in a triplex reaction) [37]. Specifically, custom FAM-labeled miR-99a/let-7c/miR-125b assay (dHsaCNS694336600) was normalized with two HEX-labeled assays as follows: RBM11 (dHsaCP2506724) targeting the centromere locus on chromosome 21 and AP3B1 (dHsaCP2500348) mapping on 5q14.1 (Bio-Rad). Briefly, the ddPCR reaction including 20 ng of gDNA, Bio-Rad ddPCR supermix for Probes No dUTP and the labeled TaqMan assays (miR-99a/let-7c/miR-125b locus assay at 100% of concentration, RBM11 100% and AP3B1 50%) were converted in approximately 15,000 droplets using the QX200 Droplet Generator and amplified according to the Bio-Rad standard protocol (60°C as annealing/extension temperature). The amplified samples were then acquired by QX100 Droplet Reader and the data were analyzed using QuantaSoft^TM^ Analysis Pro software *v.*1.0.596 (Bio-Rad). Each sample was run in duplicate with a wild type sample and negative control (no template) and the CNV of the miR-99a/let-7c/miR-125b locus was calculated as the ratio between the concentration (copies/μl) of the miR-99a/let-7c/miR-125b locus and the mean concentration value of *RBM11* and *AP3B1*, multiplied by 2. Data were obtained as the result of two technical replicate wells. CNV values higher than 2.5 or lower than 1.5 were defined as amplified or deleted locus.

### Statistical analysis

Class-comparison analysis between MPM/NC and SS/LS was performed using a receiver operating characteristic (ROC) curve and the non-parametric Mann-Whitney U test. Survival curves were plotted using the Kaplan–Meier method, and survival differences were assessed by the log-rank test using the median of each individual miRNA as a cutoff. Univariate or multivariate Cox proportional hazards were calculated considering individual miRNA as a continuous variable. Multivariate analysis was adjusted by age and histological subtype. All calculations were performed using SPSS Statistics package v.15.0.

## SUPPLEMENTARY MATERIALS FIGURES AND TABLES





## Abbreviations

CNV: copy number variation
ddPCR: droplet digital polymerase change reaction
EPP: extra-pleural pneumonectomy
FFPE: formalin-fixed paraffin-embedded
gDNA: genomic DNA
LS: long survivor
MPM: malignant pleural mesothelioma
NC: normal control
NSCLC: non-small cell lung cancer
P/D: pleurectomy with decortication
qPCR: quantitative polymerase change reaction
SS: short survivor
TS: training Set
VS: validation set.
