# Supplementary material for: Downregulation of miR-99a/let-7c/miR-125b miRNA cluster predicts clinical outcome in patients with unresected malignant pleural mesothelioma
Source: Oncotarget. 2017 Aug 2;8(40):68627–40. doi: 10.18632/oncotarget.19800 (PMC5620283; doi:10.18632/oncotarget.19800)
Supplement: Supplementary file 1 [file oncotarget-08-68627-s001.pdf]

Downregulation of miR-99a/let-7c/miR-125b miRNA cluster predicts clinical outcome in patients with unresected malignant pleural mesothelioma

SUPPLEMENTARY MATERIALS

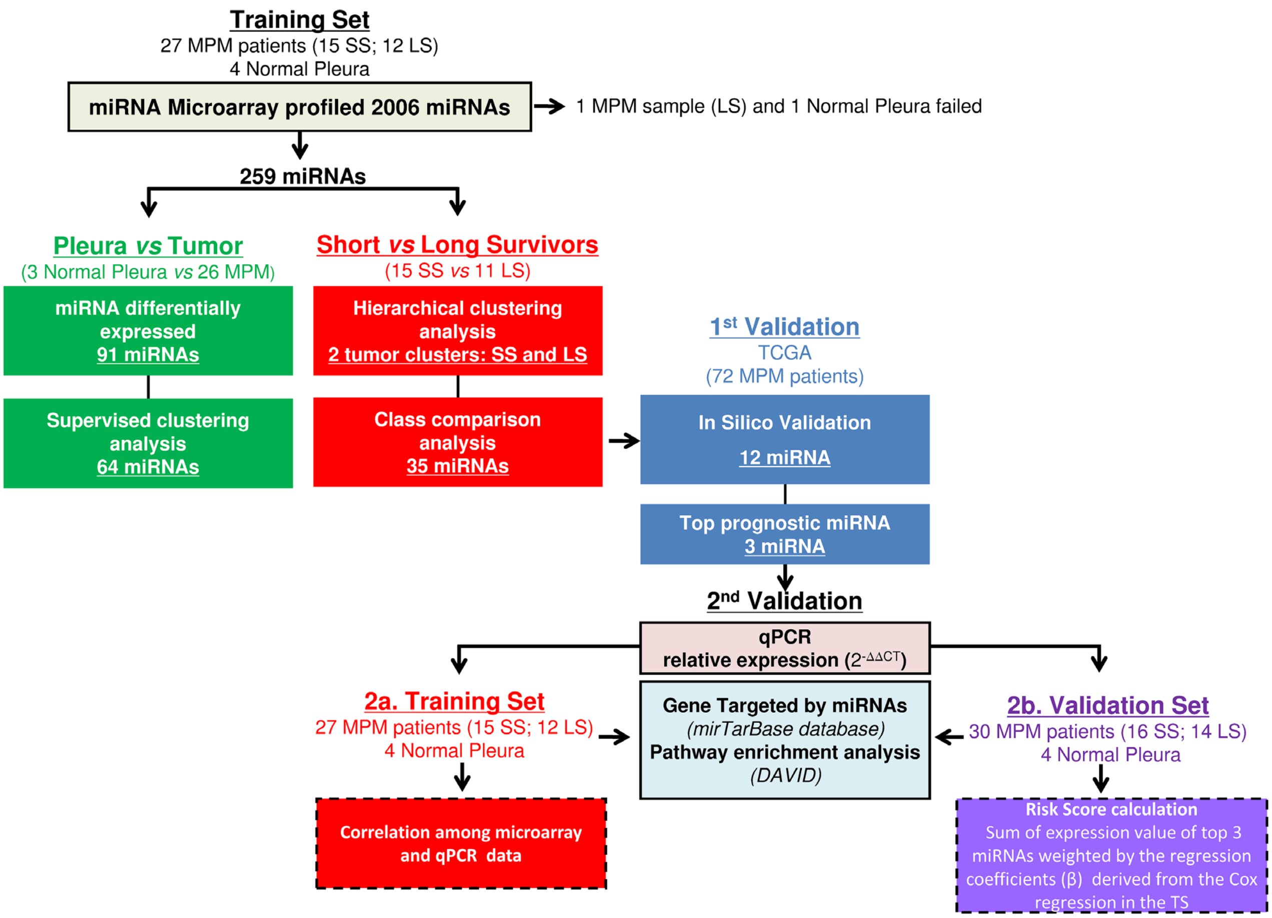

Supplementary Figure 1: Flow chart showing the specific analysis and the number of patients involved in each step.

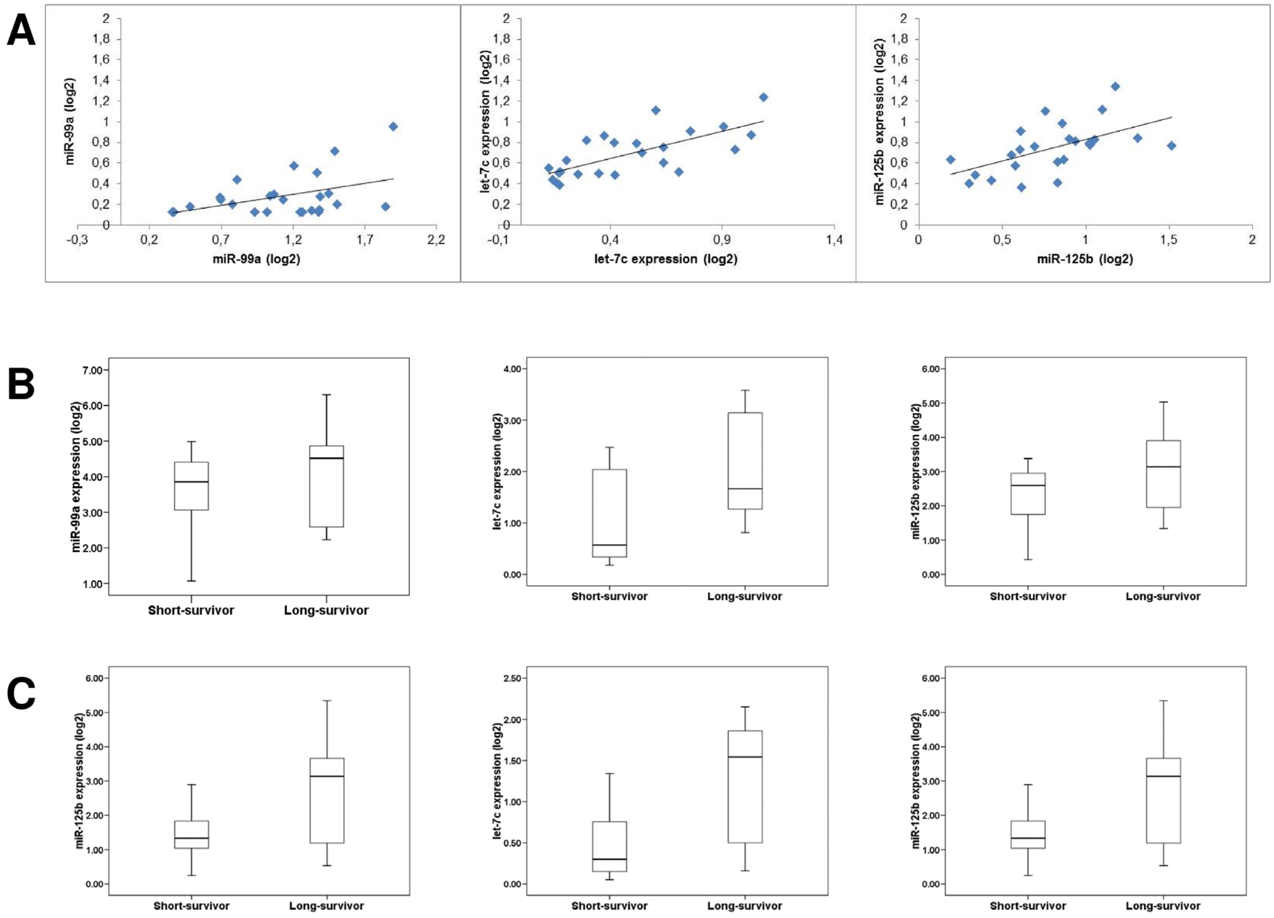

Supplementary Figure 2: (A) Scatter plots showing the correlation among the expression values of miR-99a let-7c, and miR-125b comparing microarray data with qPCR results. **(B)** Box Plots of relative expression values of miR-99a, let-7c, and miR-125b obtained by qPCR in the training set. **(C)** Box Plots of relative expression values of miR-99a, let-7c, and miR-125b obtained by qPCR in the Validation Set.

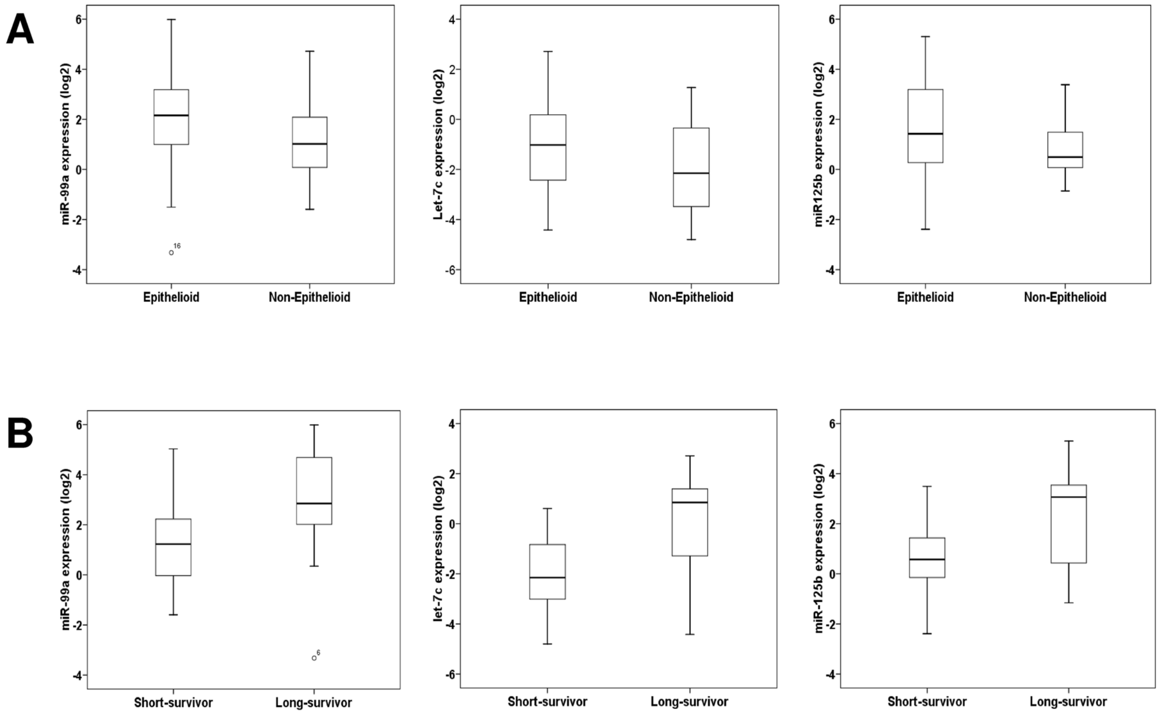

Supplementary Figure 3: (A) Box plots showing the expression of miR-99a, let-7c, and miR-125b according to the histologic type of mesothelioma. **(B)** Box plots showing the expression of miR-99a, let-7c, and miR-125b according to the survival time.

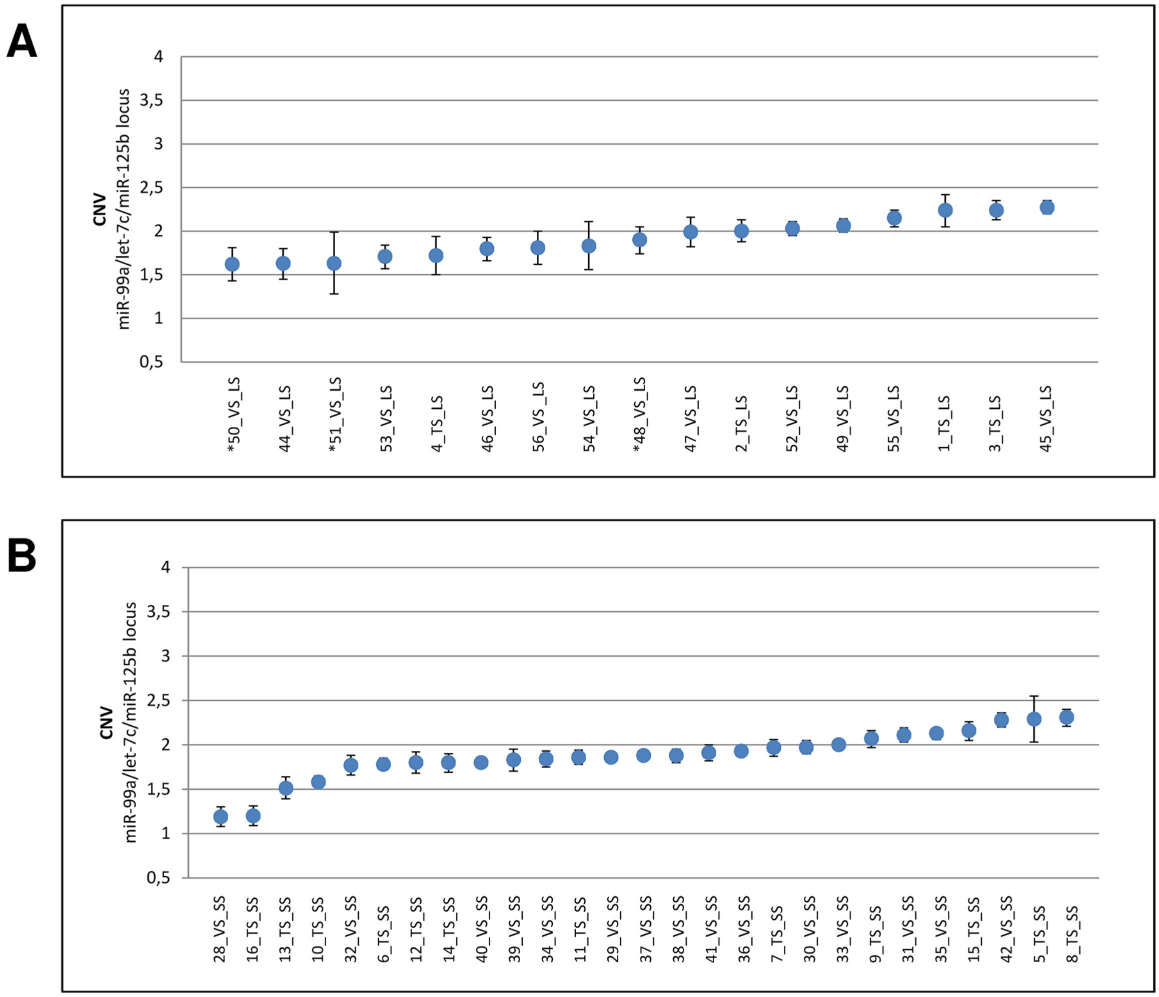

Supplementary Figure 4: Analysis of CNVs at miR-99a/let-7c/miR-125b locus in LS and SS patients. - The status of 21q21.1 locus was assessed by ddPCR normalizing the data with two reference genes (*RBM11* and *AP3B1*). The scatter plots show the copy number of locus 21q21.1 and the Poisson distribution at 95% confidence interval in 17 LS **(A)** and 27 SS **(B)** patients. The data show normal diploid status (mean value=1.89) of miR-99a/let-7c/miR-125b locus in MPM population, except to two SS patients (28_VS_SS and 16_TS_SS) reporting values (1.19 and 1.20) linked to a loss of a copy at 21q21.1 locus. However, the normalization using only the gene targeting the own centromere (*RBM11*; 21), showed normal diploid ratio (2.35 and 1.97) leading to hypothesize an unbalance between the two chromosomes rather than a loss of a copy of the locus. The miR-99a/let-7c/miR-125b locus copy number was calculated as ratio between the concentration (copies/µl) of the 21q21.1 locus and the mean concentration value of *RBM11* and *AP3B1*, multiplied by 2. Each data was obtained by merging of two technical replicate wells except 3 tumor samples (*) with insufficient starting gDNA; specifically, these samples were amplified with gDNA input lower than 10 ng (range: 4.3-8.5 ng).

Supplementary Table 1: Univariate Cox regression model hazard ratios (HR) of selected miRNAs in the training set and in the TCGA data

ID	Location	Training set		TCGA MPM dataset	
		Cox HR	Cox p-value	Cox HR	Cox p-value
**miR-99a-5p**	**21q21.1**	0.42	0.0014	0.75	0.0040
**let-7c**	**21q21.1**	0.32	0.0014	0.79	0.0240
**miR-125b-5p**	11q24.1; **21q21.1**	0.41	0.0010	0.63	0.0010
miR-26b-5p	2q35	0.45	0.0022	0.7	0.0360
miR-371a-5p	19q13.42	42.57	0.0010	2.59	0.0087
miR-23b-3p	9q22.32	0.53	0.0036	0.59	0.0060
miR-107	10q32.31	0.27	0.0040	0.67	0.0378
miR-26a-5p	3p22.2; 12q14.1	0.50	0.0041	0.59	0.0060
miR-30b-5p	8q24.22	0.46	0.0045	0.52	0.0010
miR-1185-1-3p	14q32.1	11.73	0.0050	1.81	0.0204
miR-29c-3p	1q32.2	0.56	0.0120	0.55	0.0010
miR-342-3p	14q32.2	0.40	0.0232	0.68	0.0290

Supplementary Table 2: Output of enrichment pathway analysis based on the genes targeted by miR-99a, let-7c, and miR-125b. A DAVID pathway analysis was performed using the combined list of validated targets of these miRNAs

See Supplementary File 1
