# Supplementary material for: Downregulation of miR-99a/let-7c/miR-125b miRNA cluster predicts clinical outcome in patients with unresected malignant pleural mesothelioma
Source: Oncotarget. 2017 Aug 2;8(40):68627–40. doi: 10.18632/oncotarget.19800 (PMC5620283; doi:10.18632/oncotarget.19800)
Supplement: Supplementary file 2 [file oncotarget-08-68627-s002.docx]

| **Term** | **Count** | **%** | **P-value** | **Bonferroni** | **Benjamini** | **FDR** | **Genes** |
| --- | --- | --- | --- | --- | --- | --- | --- |
| hsa05206:MicroRNAs in cancer | 45 | 4.3 | 1.9E-07 | 5.2E-05 | 5.2E-05 | 2.5E-04 | *E2F2, CYP24A1, E2F3, FGFR3, MCL1, ERBB3, ERBB2, EZH2, DICER1, BMPR2, IGF2BP1, BCL2L2, RDX, ITGB3, CCNG1, TRIM71, CCNE1, BAK1, HNRNPK, CDKN2A, BCL2, PAK4, THBS1, BMF, MYC, CREBBP, SOCS1, TP53, RAF1, HMGA2, SIRT1, BCL2L11, STAT3, NOTCH2, NRAS, CCND1, CDKN1A, SLC7A1, PLCG2, DNMT1, ABCC1, MDM4, MTOR, ABL1, CRK.* |
| hsa05220:Chronic myeloid leukemia | 18 | 1.7 | 4.6E-06 | 1.2E-03 | 6.2E-04 | 6.1E-03 | *E2F2, E2F3, TGFBR1, PIK3CD, CBL, TP53, SMAD4, RAF1, RB1, BCL2L1, AKT1, NRAS, CDKN1A, CCND1, CDKN2A, ABL1, MYC, CRK.* |
| hsa04550:Signaling pathways regulating pluripotency of stem cells | 26 | 2.5 | 7.1E-06 | 1.9E-03 | 6.3E-04 | 9.3E-03 | *FGFR2, SMARCAD1, FGFR3, BMPR2, REST, AKT1, LIF, IGF1R, ACVR1B, PCGF3, HAND1, MYC, FZD9, SETDB1, FZD8, DVL3, JARID2, PIK3CD, SMAD4, RAF1, FZD4, STAT3, NRAS, ID1, MAPK14, BMPR1B.* |
| hsa04066:HIF-1 signaling pathway | 21 | 2.0 | 7.5E-06 | 2.0E-03 | 5.0E-04 | 9.9E-03 | *IL6, ERBB2, CREBBP, EDN1, PIK3CD, MKNK2, HK2, IL6R, RPS6, STAT3, AKT1, IGF1R, CUL2, CDKN1A, EIF4EBP1, EIF4E, BCL2, PLCG2, SERPINE1, MTOR, EPO.* |
| hsa05219:Bladder cancer | 13 | 1.3 | 1.1E-05 | 3.1E-03 | 6.1E-04 | 1.5E-02 | *NRAS, E2F2, CDKN1A, E2F3, CCND1, CDKN2A, FGFR3, ERBB2, TP53, RAF1, RB1, THBS1, MYC.* |
| hsa05205:Proteoglycans in cancer | 32 | 3.1 | 1.2E-05 | 3.2E-03 | 5.4E-04 | 1.6E-02 | *ERBB3, ERBB2, PPP1R12C, RDX, ITGB3, AKT1, IGF1R, PTK2, ANK1, THBS1, MYC, ACTB, FZD9, FZD8, PIK3CD, CBL, TP53, RAF1, IGF2, RPS6, FZD4, PPP1CB, FLNB, STAT3, SMO, NRAS, CCND1, CDKN1A, PPP1CA, MAPK14, PLCG2, MTOR.* |
| hsa05223:Non-small cell lung cancer | 15 | 1.4 | 1.6E-05 | 4.3E-03 | 6.1E-04 | 2.1E-02 | *E2F2, E2F3, ERBB2, PIK3CD, TP53, RAF1, FOXO3, RB1, STK4, AKT1, NRAS, CCND1, CDKN2A, PLCG2, RARB.* |
| hsa04068:FoxO signaling pathway | 24 | 2.3 | 3.2E-05 | 8.5E-03 | 1.1E-03 | 4.2E-02 | *IRS4, IL6, ATG12, PRKAG1, TGFBR1, CREBBP, PIK3CD, SMAD4, RAF1, FOXO3, STK4, SIRT1, IL10, BCL2L11, STAT3, SOD2, AKT1, IGF1R, NRAS, CDKN1A, CCND1, S1PR1, CCNB2, MAPK14.* |
| hsa05200:Pathways in cancer | 48 | 4.6 | 9.4E-05 | 2.5E-02 | 2.8E-03 | 1.2E-01 | *FGFR2, E2F2, E2F3, FGFR3, XIAP, ERBB2, BCL2L1, GLI1, AKT1, CCNE1, IGF1R, CUL2, PTK2, CDKN2A, RASGRP1, BCL2, PLEKHG5, RARB, MYC, GNG5, FZD9, CEBPA, DVL3, FZD8, IL6, COL4A1, TGFBR1, CREBBP, PIK3CD, CBL, TP53, SMAD4, RAF1, ITGA3, RB1, STK4, FZD4, STAT3, SMO, NRAS, CCDC6, CCND1, CDKN1A, ADCY9, PLCG2, MTOR, ABL1, CRK.* |
| hsa04012:ErbB signaling pathway | 17 | 1.6 | 2.2E-04 | 5.8E-02 | 6.0E-03 | 2.9E-01 | *ERBB3, ERBB2, PIK3CD, CBL, RAF1, AKT1, NRAS, PTK2, CDKN1A, EIF4EBP1, PAK4, PLCG2, MTOR, ABL1, MAP2K7, MYC, CRK.* |
| hsa05215:Prostate cancer | 17 | 1.6 | 2.6E-04 | 6.6E-02 | 6.2E-03 | 3.4E-01 | *FGFR2, E2F2, E2F3, ERBB2, PIK3CD, CREBBP, TP53, RAF1, RB1, AKT1, CCNE1, IGF1R, NRAS, CDKN1A, CCND1, BCL2, MTOR.* |
| hsa05230:Central carbon metabolism in cancer | 14 | 1.4 | 3.1E-04 | 8.0E-02 | 7.0E-03 | 4.1E-01 | *FGFR2, FGFR3, ERBB2, PIK3CD, TP53, HK2, RAF1, PFKM, NTRK3, AKT1, PKM, NRAS, MTOR, MYC.* |
| hsa05214:Glioma | 14 | 1.4 | 3.7E-04 | 9.4E-02 | 7.5E-03 | 4.8E-01 | *E2F2, E2F3, PIK3CD, TP53, RAF1, RB1, AKT1, IGF1R, NRAS, CCND1, CDKN1A, CDKN2A, PLCG2, MTOR.* |
| hsa05212:Pancreatic cancer | 14 | 1.4 | 3.7E-04 | 9.4E-02 | 7.5E-03 | 4.8E-01 | *E2F2, E2F3, ERBB2, TGFBR1, PIK3CD, TP53, SMAD4, RAF1, BCL2L1, RB1, STAT3, AKT1, CCND1, CDKN2A.* |
| hsa05222:Small cell lung cancer | 16 | 1.5 | 5.5E-04 | 1.4E-01 | 1.1E-02 | 7.2E-01 | *E2F2, E2F3, COL4A1, XIAP, PIK3CD, TP53, ITGA3, BCL2L1, RB1, AKT1, CCNE1, PTK2, CCND1, BCL2, RARB, MYC.* |
| hsa05012:Parkinson's disease | 22 | 2.1 | 5.9E-04 | 1.5E-01 | 1.1E-02 | 7.8E-01 | *ND1, ATP5E, NDUFA2, ND4, ND5, ND2, ATP5B, ND3, COX7C, VDAC1, NDUFS7, GNAL, SDHB, UQCR10, ND4L, COX3, COX2, COX1, COX6B1, UBB, ND6, ATP6.* |
| hsa00190:Oxidative phosphorylation | 21 | 2.0 | 6.4E-04 | 1.6E-01 | 1.1E-02 | 8.4E-01 | *ND1, ATP5E, NDUFA2, ND4, ND5, ND2, ATP5B, ND3, COX7C, ATP6V1G1, ATP6V1F, NDUFS7, SDHB, UQCR10, ND4L, COX3, COX2, COX1, COX6B1, ND6, ATP6.* |
| hsa04115:p53 signaling pathway | 13 | 1.3 | 1.7E-03 | 3.7E-01 | 2.6E-02 | 2.2E+00 | *TP53, PMAIP1, CCNG1, CCNE1, CDKN1A, CCND1, CDKN2A, CCNB2, BBC3, RRM2, SERPINE1, MDM4, THBS1.* |
| hsa04110:Cell cycle | 19 | 1.8 | 1.8E-03 | 3.8E-01 | 2.6E-02 | 2.3E+00 | *E2F2, YWHAZ, E2F3, E2F5, CREBBP, TP53, SMAD4, ESPL1, RB1, WEE1, CCNE1, CDKN1A, CCND1, CDKN2A, CCNB2, ORC5, ABL1, SMC1A, MYC.* |
| hsa05161:Hepatitis B | 21 | 2.0 | 1.9E-03 | 4.0E-01 | 2.7E-02 | 2.5E+00 | *E2F2, IL6, YWHAZ, E2F3, TGFBR1, CREBBP, PIK3CD, TP53, SMAD4, RAF1, RB1, STAT3, STAT2, AKT1, CCNE1, NRAS, CDKN1A, CCND1, DDX3X, BCL2, MYC.* |
| hsa03010:Ribosome | 20 | 1.9 | 2.1E-03 | 4.3E-01 | 2.8E-02 | 2.7E+00 | *RPL35A, RPL36A, RPS6, RPL29, RPS7, RPS26, MRPL12, RPS28, RPL18A, RPS3A, RPLP0, RPL9, RPS15, RPL3, RPS12, RPS4Y1, RPS13, RPL37A, UBA52, RPS24.* |
| hsa03013:RNA transport | 23 | 2.2 | 3.1E-03 | 5.6E-01 | 3.8E-02 | 4.0E+00 | *EEF1A1, SNUPN, PABPC4, EIF5, NUP93, CASC3, NUP155, FXR1, NDC1, EIF3C, EIF4A3, EIF4G2, SUMO1, EIF4EBP1, EIF3A, EIF4E, NUP205, POP1, SEC13, NUP37, RANBP2, PABPC1, KPNB1.* |
| hsa03015:mRNA surveillance pathway | 15 | 1.4 | 3.2E-03 | 5.8E-01 | 3.9E-02 | 4.2E+00 | *PABPC4, HBS1L, CASC3, ETF1, CSTF2T, PPP1CB, EIF4A3, PCF11, PPP1CA, PPP2CA, NUDT21, MSI2, PPP2R5E, PABPC1, PPP2R2A.* |
| hsa04152:AMPK signaling pathway | 18 | 1.7 | 3.7E-03 | 6.2E-01 | 4.2E-02 | 4.7E+00 | *IRS4, SREBF1, PRKAG1, PIK3CD, ADIPOR2, FOXO3, PFKM, SIRT1, AKT1, IGF1R, CCND1, EIF4EBP1, PPP2CA, PPP2R5E, MTOR, RAB10, TBC1D1, PPP2R2A.* |
| hsa04919:Thyroid hormone signaling pathway | 17 | 1.6 | 4.4E-03 | 6.9E-01 | 4.8E-02 | 5.6E+00 | *ACTB, PIK3CD, CREBBP, TP53, MED12, RAF1, ATP1A1, ITGB3, AKT1, NOTCH2, NRAS, CCND1, NCOA3, PLCG2, MTOR, MYC, MED1.* |
